# Identification of Tick *Ixodes ricinus* Midgut Genes Differentially Expressed During the Transmission of *Borrelia afzelii* Spirochetes Using a Transcriptomic Approach

**DOI:** 10.3389/fimmu.2020.612412

**Published:** 2021-02-04

**Authors:** Sazzad Mahmood, Radek Sima, Veronika Urbanova, Jos J. A. Trentelman, Nicolas Krezdorn, Peter Winter, Petr Kopacek, Joppe W. Hovius, Ondrej Hajdusek

**Affiliations:** ^1^ Institute of Parasitology, Biology Centre, Czech Academy of Sciences, Ceske Budejovice, Czechia; ^2^ Faculty of Science, University of South Bohemia, Ceske Budejovice, Czechia; ^3^ Center for Experimental and Molecular Medicine, Amsterdam Infection and Immunity Institute, Amsterdam UMC, Location Academic Medical Center, University of Amsterdam, Amsterdam, Netherlands; ^4^ GenXPro GmbH, Frankfurt Innovation Center Biotechnology, Frankfurt am Main, Germany

**Keywords:** *Borrelia afzelii*, *Ixodes ricinus*, transcriptome, tick, midgut, RNAi, massive analysis of cDNA ends (MACE)

## Abstract

Lyme borreliosis is an emerging tick-borne disease caused by spirochetes *Borrelia burgdorferi* sensu lato. In Europe, Lyme borreliosis is predominantly caused by *Borrelia afzelii* and transmitted by *Ixodes ricinus*. Although *Borrelia* behavior throughout tick development is quite well documented, specific molecular interactions between *Borrelia* and the tick have not been satisfactorily examined. Here, we present the first transcriptomic study focused on the expression of tick midgut genes regulated by *Borrelia*. By using massive analysis of cDNA ends (MACE), we searched for tick transcripts expressed differentially in the midgut of unfed, 24h-fed, and fully fed *I. ricinus* nymphs infected with *B. afzelii*. In total, we identified 553 upregulated and 530 downregulated tick genes and demonstrated that *B. afzelii* interacts intensively with the tick. Technical and biological validations confirmed the accuracy of the transcriptome. The expression of five validated tick genes was silenced by RNA interference. Silencing of the uncharacterized protein (GXP_Contig_30818) delayed the infection progress and decreased infection prevalence in the target mice tissues. Silencing of other genes did not significantly affect tick feeding nor the transmission of *B. afzelii*, suggesting a possible role of these genes rather in *Borrelia* acquisition or persistence in ticks. Identification of genes and proteins exploited by *Borrelia* during transmission and establishment in a tick could help the development of novel preventive strategies for Lyme borreliosis.

## Introduction

Lyme borreliosis is an emerging human disease, occurring predominantly in temperate regions of the northern hemisphere (1, 2). It is caused by spirochetes *Borrelia burgdorferi* sensu lato and is spread by ticks from the genus *Ixodes*. In Europe, ~65,000 new cases are reported annually (3). However, the real prevalence is substantially higher due to under-reporting (4). In North America, the transmission cycle primarily involves the spirochete *B. burgdorferi* sensu stricto and the tick *Ixodes scapularis*. In Europe, the disease is caused by several *Borrelia* species and is transmitted by related tick species, *Ixodes ricinus* and *Ixodes persulcatus*. The early disease typically manifests itself with a bulls-eye rash on the skin, called *erythema migrans*. The spirochetes then disseminate throughout the body to diverse tissues and are associated with arthritis, neurological symptoms, and dermatitis (5). Prompt antibiotic treatment usually cures the disease and symptoms. Despite several promising trials (6–9), a vaccine against human Lyme borreliosis is not currently available and prevention mainly depends on avoiding tick bites (10).

*Ixodes ricinus* is the most common tick in Europe and is typically found in humid sheltered environments and forests, mainly from early spring until late fall. It is a three-host tick, where all developmental stages (larva, nymph, and adult female) must feed on the host blood to undergo molting into the next instar. *B. afzelii* is the dominant spirochete in Europe (11). *Borrelia* enter the tick gut when the larvae feed on an infected mouse. The spirochetes then multiply and are transstadially maintained in the tick through the molts (12). The nymph’s ability to survive without feeding for years contributes to stabilization of *Borrelia* prevalence in the reservoir host population. Because of their small size, the tick nymphs are considered to be the most critical tick stage for human infections (13). During engorgement, which typically lasts for two to four days, the spirochetes continuously migrate from the tick into the host. An interval between 24 and 48 h after tick attachment is considered the most critical time for transition of *B. afzelii*. Although *Borrelia* can already be detected in the skin on the first day of feeding, this early spirochetal population cannot initiate a systemic infection (12). Unlike *B. burgdorferi* s.s. in *I. scapularis* (14), which migrate through the hemolymph and salivary glands into the host, *B. afzelii* probably infect the host directly from the midgut of *I. ricinus* (12).

The segmented tick midgut is well adapted to accommodate an enormous volume of host blood. Unlike other blood-feeding arthropods, digestion in ticks occurs intracellularly (15), so extracellular pathogens are not directly exposed to the harsh effects of secreted proteases. Despite this, the tick midgut is still a relatively sterile environment (16), maintained presumably by combining active components of the blood and tick immune molecules. Adaptations of *Borrelia* spirochetes inhabiting the tick midgut are still not satisfactorily explained. However, it has been documented that during tick colonization, *Borrelia* change expression of their genes (17). For instance, the main surface protein outer surface protein A (OspA) is preferentially expressed within the tick midgut and is downregulated during transmission of the spirochete to the vertebrate host (18). The tick receptor for OspA (TROSPA), is a midgut protein identified in *Ixodes scapularis*, ensuring adherence of *B. burgdorferi* to the midgut surface. Expression of *trospa* is significantly upregulated in *Borrelia*-infected nymphs. Moreover, the silencing of *trospa* expression reduces colonization and transmission of the pathogen (19). Another example of this co-adaptation is *Borrelia*-induced overexpression of the tick salivary protein 15 (Salp15) necessary for *Borrelia* survival in the host (20). *Borrelia*-infected nymphs have also been shown to accumulate significantly more fat reserves (21) to better survive unfavorable temperatures and humidities (22). These examples point to the existence of delicate gene interactions between *Borrelia* spirochetes and the tick.

Here we show that midgut cells of infected nymphs before, during, and after feeding on the vertebrate host react to *B. afzelii*. By employing the MACE transcriptomic method, we were able to identify, in total, 1,083 *Borrelia*-responding tick midgut genes. Silencing of tick uncharacterized protein (GXP_Contig_30818) by RNA interference reduced transmission of *Borrelia* spirochetes from the tick to the host, whereas silencing of several other candidate tick genes had no effect. This suggests that these genes may have a role in processes associated with acquisition rather than transmission of *Borrelia*, and persistence in the vector.

## Material and Methods

### Biological Material

**Figure 1:**
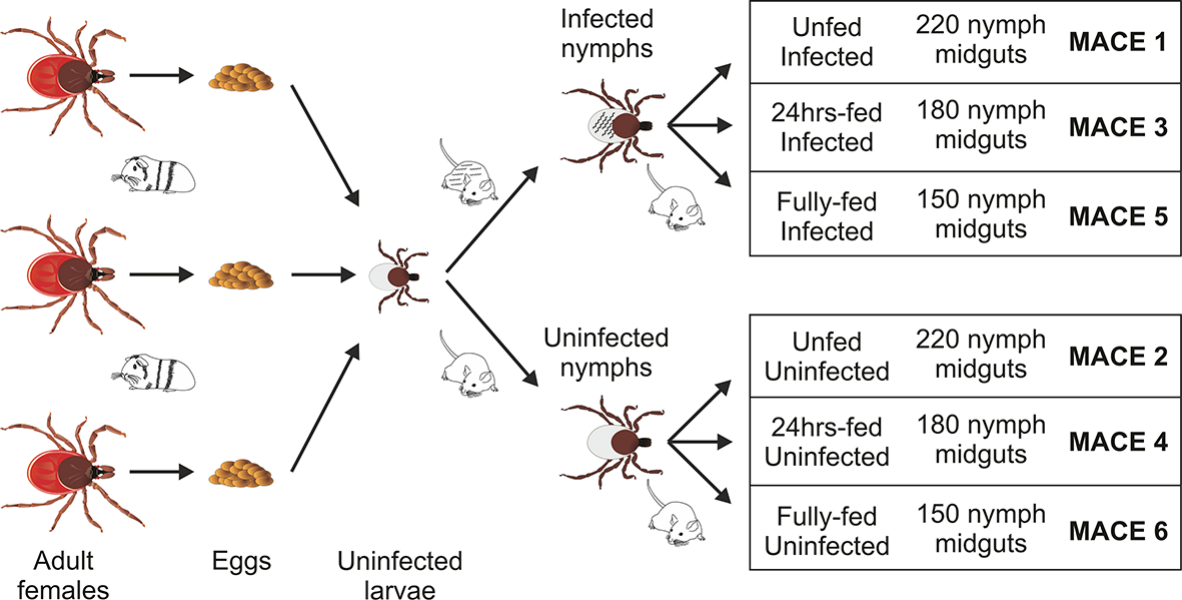
Scheme of sample preparation for massive analysis of cDNA ends (MACE) analysis. The uninfected larvae, originating from three individual females fed on a single guinea pig, were mixed and fed on *B. afzelii*-infected or uninfected mice. The nymphs then were fed on uninfected mice and dissected for midguts (150–220 nymphs for each group) at the three indicated time points. The MACE analyses were performed on six different RNA pools (MACE 1-6).

Adult females of *I. ricinus* were collected by flagging in a forest near Ceske Budejovice and kept at 95% humidity, 24°C, and 15/9 daylight settings. The adult ticks were fed on a single guinea pig. The laid eggs were preserved to hatch separately to form individual populations, each coming from a single female. For the transcriptomics purposes, the larvae from three populations were mixed together to scale up the number of ticks and then divided into two groups to prepare for infected and uninfected nymphs (**Figure 1**). Prior to feeding, a half of 6–8 week old C3H/HeN mice (Charles River Laboratories, GER) were infected with *B. afzeli* CB43 (23) by subcutaneous injection of 0.2 ml of culture (approximately 10^6^ spirochetes). Mouse infection was checked by PCR on ear punctures taken 3 weeks after injection. The *Borrelia*-infected nymphs were obtained by feeding the larvae on *Borrelia*-infected mice. Uninfected nymphs were obtained by feeding the larvae on uninfected C3H/HeN mice. The resulted nymphs, molted 4–6 weeks after repletion, were rested for 2 weeks and used in these experiments. The prevalence of *Borrelia* infection in nymphs was checked by PCR and reached >90%. All experiments were carried out according to the animal protection law of the Czech Republic (§17, Act No. 246/1992 Sb) with the approval of CAS (approval no. 79/2013). The experiments with *Borrelia* were performed in BSL2 conditions.

### Tick Dissection and RNA Extraction

The *Borrelia*-infected nymphs were divided into three groups (MACE 1,3,5), as well as the uninfected nymphs, which were also divided into three groups (MACE 2,4,6). The nymphs of MACE groups 1 and 2 remained unfed. The nymphs of MACE groups 3 and 4 were forcibly removed from the naïve 6–8 weeks old C3H/HeN mice at 24 h after attachment. The nymphs of MACE groups 5 and 6 were allowed to feed on the naïve 6–8 weeks old C3H mice until repletion (around 72 h). All tick were surface-sterilized by washing in 3% H_2_O_2_, 70% ethanol, and distilled water (30 seconds each wash). The nymphs were dissected for midguts [pools of: 220 unfed nymphs (MACE 1,2), 180 24 h-fed nymphs (MACE 3,4), and 150 fully fed nymphs (MACE 5,6)] under the stereomicroscope (Olympus) on wax dishes with diethyl pyrocarbonate (DEPC)-treated cold phosphate buffered saline (PBS) and then transferred in RA1 buffer (NucleoSpin miRNA Kit, Macherey-Nagel, GER) supplemented with β-mercaptoethanol (Sigma-Aldrich). Before extraction, the midguts were homogenized in an insulin syringe. Total RNA (including miRNA) was extracted using the above extraction kit by following the manufacturer’s protocol (“small+large” protocol). The concentration of RNA was measured by NanoDrop ND-1000 (Thermo Fisher Scientific), its consistency was checked on an agarose gel, and stored at −80°C until further use.

### MACE Analysis

The massive analysis of cDNA ends (MACE) was performed as previously described (24) using the GenXPro MACE Kit (GenXPro) according to the manufacturer’s protocol (www.genxpro.net). The isolated RNA was subjected to an additional DNAse I treatment and its quality was assessed on an Agilent 2100 Bioanalyzer. First and second-strand cDNA synthesis was then performed, initiated from biotinylated oligo dT primers. The cDNA was fragmented randomly by ultrasonication, resulting in fragments with an average size of 300bp as determined by an Agilent 2100 Bioanalyzer. The biotinylated 3′ cDNA ends were bound to a streptavidin matrix, while the remaining fragments were eliminated through the washing step. Then, the p5 “TrueQuant” sequencing adapter was ligated to the unbound end of the fragments using tailed Illumina p5 and p7 oligonucleotides as primers. The quality of the final library was determined using an Agilent 2100 Bioanalyzer. The next-generation single-end sequencing of the 5’ cDNA fragments was performed on an Illumina HiSeq2000 sequencer. To remove the PCR bias, all duplicate reads detected by the GenXPro in-house TrueQuant technology were removed from the raw datasets. In addition, low-quality sequence nucleotides and poly(A)-tails were clipped off using Cutadapt (25). Overlapping sequencing reads were then assembled into contigs. The reads were aligned to different reference sequences using NovoAlign (www.novocraft.com/products/novoalign/), resulting in “GXP_Contigs” (sequences derived from our previously published nymphal RefSeq database (Bioproject PRJNA657487), “Contigs” (*I. ricinus* sequences were derived from NCBI nuccore and the BioProjects 177622, 230499, 34667, and 183509), and “noHITAssemblies” (assemblies of MACE sequences that could not be mapped to sequences from the existing BioProjects or our own RefSeq database). The contigs of the assemblies were annotated further by BLASTX to either the SwissProt or Trembl database (www.uniprot.org). Contigs that did not match to one of these databases were annotated by BLASTN to all “*Ixodes*” mRNA sequences available in the NCBI database, against the “nt” (nucleotide collection from GenBank, RefSeq, TPA, and PDB) of NCBI, or the *I. scapularis* genome (NW_002505054). Only uniquely mapped reads were accepted for the quantification of the MACE tags. Finally, gene expression was normalized per million reads and tested for differential gene expression between the different conditions using the DEGSeq R/Bioconductor package (26) (R package version 1.16.0.). The final table was produced as an Excel file (**Supplemental Table 1**).

### *In Silico* Analysis

The selection of *Borrelia*-upregulated and downregulated genes at different time points was performed using the MACE Excel file according to these selection criteria: 1) the transcript was annotated (e-value ≤ 10E^−6^) in the *I. ricinus* genome PRJNA270959, the *I. scapularis* genome PRJNA314100, or in all *Ixodes* sequences available in NCBI; 2) “noHitAssemblies” contigs were removed from the analysis because of no homologies with tick sequences (no hits, host contaminants, and short-length sequences); 3) to select upregulated genes: fold change upregulation of expression in the infected *vs*. uninfected group was set to ≥ 5 and expression in the infected group to ≥ 5 transcripts per million; selection of downregulated genes was done *vice versa* (expression in the uninfected *vs*. infected group was set to ≥ 5 and expression in the uninfected group to ≥ 5 transcripts per million). The selected candidate sequences were translated into proteins (DNASTAR) and screened for the presence of a signal sequence by SignalP 5.0 (www.cbs.dtu.dk/services/SignalP/) and for cellular localization by DeepLoc-1.0 (www.cbs.dtu.dk/services/DeepLoc/).

### Technical and Biological Validation of the MACE Analysis

An aliquot of RNA from each MACE analysis was used for the technical validation of MACE results. For biological validation, we prepared 10 genetically distinct larval populations of *I. ricinus* ticks coming from wild-captured adult females fed on a guinea pig (**Supplemental Figure 1**). Each of the batches of larvae was divided in half and fed on *B. afzelii* CB43-infected or uninfected 6–8 weeks old C3H/HeN mice (Charles River Laboratories, GER) mice. The resulting infected and uninfected nymphs were then fed on naïve mice for 0h, 24h, and until replete (fully fed), midguts were dissected (for each group and time point pools of: 50 unfed nymphs, 20 24h-fed nymphs, and 10 fully fed nymphs (equal number of females and males), and RNA was extracted following the methods and time points used for the MACE analysis. Then, the RNA was reverse transcribed into cDNA (0.5µg RNA per 20µl reaction; random hexamers) using the Transcriptor High-Fidelity cDNA Synthesis Kit (Roche) and diluted 20-times in sterile water. Gene-specific qRT-PCR primers were designed in Primer3 (http://bioinfo.ut.ee/primer3-0.4.0/) and verified by PCR using cDNA prepared from a mix of infected nymphs at different time points. Gene expression in technical and biological replicates was measured by quantitative real-time PCR (qRT-PCR) using a LightCycler 480 (Roche) and SYBR green chemistry, as described previously (27) and primers listed in **Supplemental Table 2**. Relative expression was normalized to *I. ricinus* elongation factor (GU074769) and ferritin 1 (AF068224, data not shown) using the mathematical model of Pfaffl (28).

### RNA Interference and Its Effect on Nymph Feeding and Development

To prepare the gene-specific dsRNA, 200–600bp long gene fragments were amplified from *I. ricinus* cDNA and cloned into the pll10 vector with two T7 promoters in reverse orientations (29), using primers listed in **Supplemental Table 2** and containing additional restriction sites ApaI and XbaI. The dsRNA was synthesized as described previously (30). The dsRNA (3 μg/μl) was injected through the coxa of the third pair of legs into the hemocoel of nymphs (32 nl) using Nanoinject II (Drummond). After 3 days of rest in a humid chamber at room temperature, the nymphs (20 nymphs per mouse, 3 mice per group) were fed on BALB/c mice (Velaz, CR). The level of gene silencing was checked by qRT-PCR in a mix of five fully fed nymphs and compared to the dsGFP control group. For each group, we recorded feeding success, length of feeding, the weight of individual nymphs after feeding, and molting into adults (took approximately 2 months; recorded every 2 weeks until molting in the dsGFP control group reached 80%).

### *Borrelia*-Transmission Experiments

*Borrelia afzelii* CB43-infected nymphs were prepared as described previously (31). The infected nymphs were injected with 32nl of gene-specific dsRNA or dsGFP (control), rested for 3 days, and fed on the uninfected 6-weeks old C3H/HeN mice (five nymphs per mouse, 5–8 mice per group) in plastic cylinders attached to the murine back. Detached engorged nymphs were weighed. The level of *Borrelia* infection in each mouse was measured the second week after tick detachment by qRT-PCR using DNA isolated from an ear biopsy and normalized to the number of mouse genomes (actin). Three weeks after tick detachment, mice were sacrificed and the numbers of *Borrelia* in the ear, urinary bladder, and heart tissue were determined by qRT-PCR as reported previously (12).

### Statistical Analysis

For biological validations, feeding experiments, and transmission experiments, statistical significance of differences were analyzed using GraphPad Prism 8.0 (GraphPad Software, CA) employing the One-way ANOVA Kruskal-Wallis test or the non-parametric Mann-Whitney test and P < 0.05 (∗), P < 0.01 (∗∗), or P < 0.001 (∗∗∗) were considered as significant. If not further specified, all results were expressed as the mean ± standard error (SEM).

## Results

### MACE Analysis

Initially, we measured differences in gene expression of *Borrelia*-infected ticks by employing the MACE technology, where high throughput sequencing of cDNA fragments provides a high resolution of gene expression and can reveal expression of low-abundance transcripts, compared to standard RNA sequencing (24, 32). We pooled more than 150 nymph midguts from each stage of tick feeding to minimize variations in gene expression. Being aware of intra-species genetic variation of wild-captured ticks, we limited the transcriptomes to the mixed population of nymphs originating from only three tick females (**Figure 1**). During the preparation of ticks for the transcriptomes and biological validations, we did not observe any adverse effects of the *Borrelia* infection on tick survival, fitness, or feeding, as demonstrated by body weights of fully fed infected nymphs compared with uninfected controls (**Supplemental Figure 2**). As a result, we obtained a total of 38,199,641 raw reads from the six cDNA MACE libraries. By mapping these sequences to our previously sequenced RefSeq library [containing 32,897 high-quality GXP contigs; Bioproject PRJNA657487 (33)] and the public *Ixodes* genomic and transcriptomic databases, we identified in each MACE library, on average, 17,257 GXP contigs and 1,302 additional tick genome/transcriptome contigs (gi|contigs absent from the RefSeq database) (**Supplemental Table 3**). Overall, in the midgut transcripts, we observed a total of 24,276 tick genes. This number is in line with the 26,179 transcripts identified in our previous MACE transcriptomic project of the nymph *I. ricinus* salivary glands (33) and lower than the total number of genes described in the tick *I. scapularis* genome project (32,572 protein-coding genes) (34).

### Identification of the Differentially Expressed Genes

**Figure 2:**
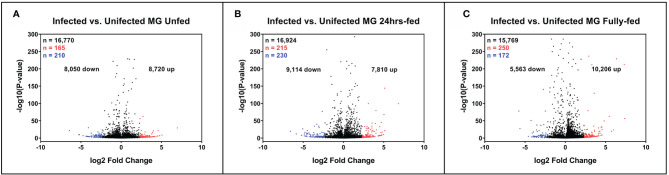
Expression of tick midgut genes is altered in the presence of *Borrelia afzelii*. Volcano plots showing differentially expressed tick transcripts analyzed by MACE at individual time points. **(A)** Unfed nymphs **(B)** Nymphs fed for 24 h **(C)** Fully fed nymphs. n = number of differentially expressed transcripts. Total differentially expressed transcripts (black), upregulated transcripts (red; p-value ≤ 0.05 and log2 fold change ≥ 2.3), and downregulated transcripts (blue; p-value ≤ 0.05 and log2 fold change ≤ −2.3). up = total upregulated transcripts, down = total downregulated transcripts, MG, midgut.

**Figure 3:**
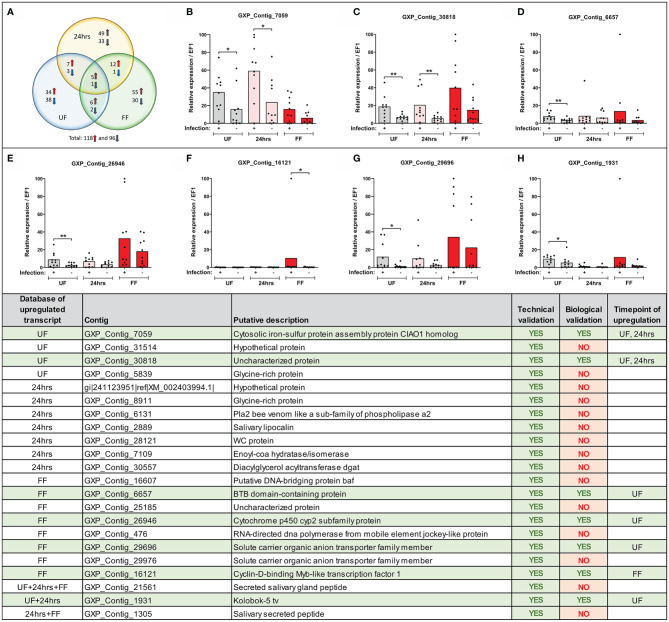
Expression of selected transcripts can be verified by technical and biological qRT-PCR validations. (Upper) **(A)** Venn diagram of the top-score differentially expressed *B. afzelii*-infected nymph midgut transcripts (fold change ≥ 5 fold and expression ≥ 5 transcripts per million). The upregulated transcripts are marked by a red arrow, downregulated by a blue arrow. **(B–H)** qRT-PCR profiles (relative expression) of seven biologically validated transcripts were significantly upregulated by *B. afzelii* infection (Mann-Whitney test). The biological validations were carried out on 10 individual tick populations. Each dot represents expression in a single nymph population. In each graph, cDNA with the highest expression was set as 100. The tick *elongation factor* was used as a housekeeping gene. (Lower) Summary table of the validated transcripts. In total, 22 transcripts from different time points of feeding (see *Results* for the selection criteria) were analyzed by the technical and biological validations. UF, unfed; 24hrs, fed for 24hrs; FF, fully fed. P < 0.05 (*), P < 0.01 (**).

To sort the database for genes upregulated or downregulated in the presence of *B. afzelii*, we defined a transcript as differentially expressed when the fold change was ≥ 5 (log2fold change ≤ −2.3 or ≥ 2.3) and the p-value ≤ 0.05. This primary selection led to the identification of 553 upregulated and 530 downregulated unique genes (**Figure 2**). Interestingly, in the group of fully fed nymphs (**Figure 2C**), we identified the largest number of *Borrelia*-upregulated genes (fold change > 1) and the highest ratio between upregulated and downregulated transcripts. Then, to produce a slimmed list of the differentially regulated genes, potentially confirmable by qRT-PCR in technical and biological validations, we selected transcripts with a fold change ≥ 5 and expression ≥ 5 transcripts per million in the infected (for upregulated genes) or uninfected (for downregulated genes) groups. By applying these criteria, we obtained a list of 118 upregulated and 96 downregulated genes (**Figure 3A**), of which 34, 49, and 55 genes were upregulated by infection at unfed (UF), 24h-fed (24-h), and fully fed (FF) stages, respectively. Conversely, 38, 33, and 30 genes were downregulated. Interestingly, five genes were upregulated, and one gene downregulated in all three time points (**Supplemental Tables 4**–**7**). The genes encode potentially secreted proteins (SignalP) containing a signal sequence [labeled as “SP(Sec/SPI)”] or intracellular proteins (labeled as “OTHER”). We did not observe any pattern in the prediction of subcellular localization (DeepLoc). The full list contained extracellular proteins, as well as proteins localized to the cytoplasm, mitochondrion, nucleus, or lysosome. Most of the proteins were predicted to be soluble, although the list also contained several membrane proteins (e.g., receptors, channels, glycoproteins). In summary, we identified 214 tick genes with various functions and localizations, highly differentially expressed in the presence of *B. afzelii*, suggesting a significant interaction of the tick midgut tissue with the spirochetes.

### Technical and Biological Validations of MACE

We confirmed the expression of several differentially regulated genes arising from the MACE analysis by technical and biological validations. We focused only on genes from our upregulated candidate list as only these could be later silenced by RNA interference and tested in our *B. afzelii*-transmission model. We selected 46 candidates (from various time points) with homologous sequences present in the genomic databases of *I. ricinus* and *I. scapularis* (for the selection criteria see *Methods*). For 33 of these genes, we were able to design gene-specific PCR primers and for 22 of these genes, these primers worked well in a standard PCR assay. Their expression was then validated in technical and biological validations by qRT-PCR. All 22 candidate genes passed the technical validation and were proven to be upregulated at specific time points (**Figure 3**). Gene expression levels in 10 genetically distinct *I. ricinus* populations of nymphs were then determined to validate these candidate genes biologically. Through this strict validation level, seven genes passed, representing 32% of the 22 pre-selected genes. Of these, four candidates were shown to be overexpressed at the same time point compared to MACE, while the other three genes were overexpressed at other time points. The seven gene sequences represented: 1) cytosolic iron-sulfur protein assembly protein CIAO1 homolog (GXP_Contig_7059), 2) uncharacterized protein (GXP_Contig_30818), 3) BTB domain-containing protein (GXP_Contig_6657), 4) cytochrome p450 cyp2 subfamily protein (GXP_Contig_26946), 5) solute carrier organic anion transporter family member (GXP_Contig_29696), 6) cyclin-D-binding Myb-like transcription factor 1 (GXP_Contig_16121), and 7) Kolobok-5 tv protein (GXP_Contig_1931). All transcripts encoded intracellular proteins without predicted signal sequences (SignalP) and were predicted for various cellular localizations (DeepLoc).

### RNA Interference and *Borrelia*-Transmission

**Figure 4:**
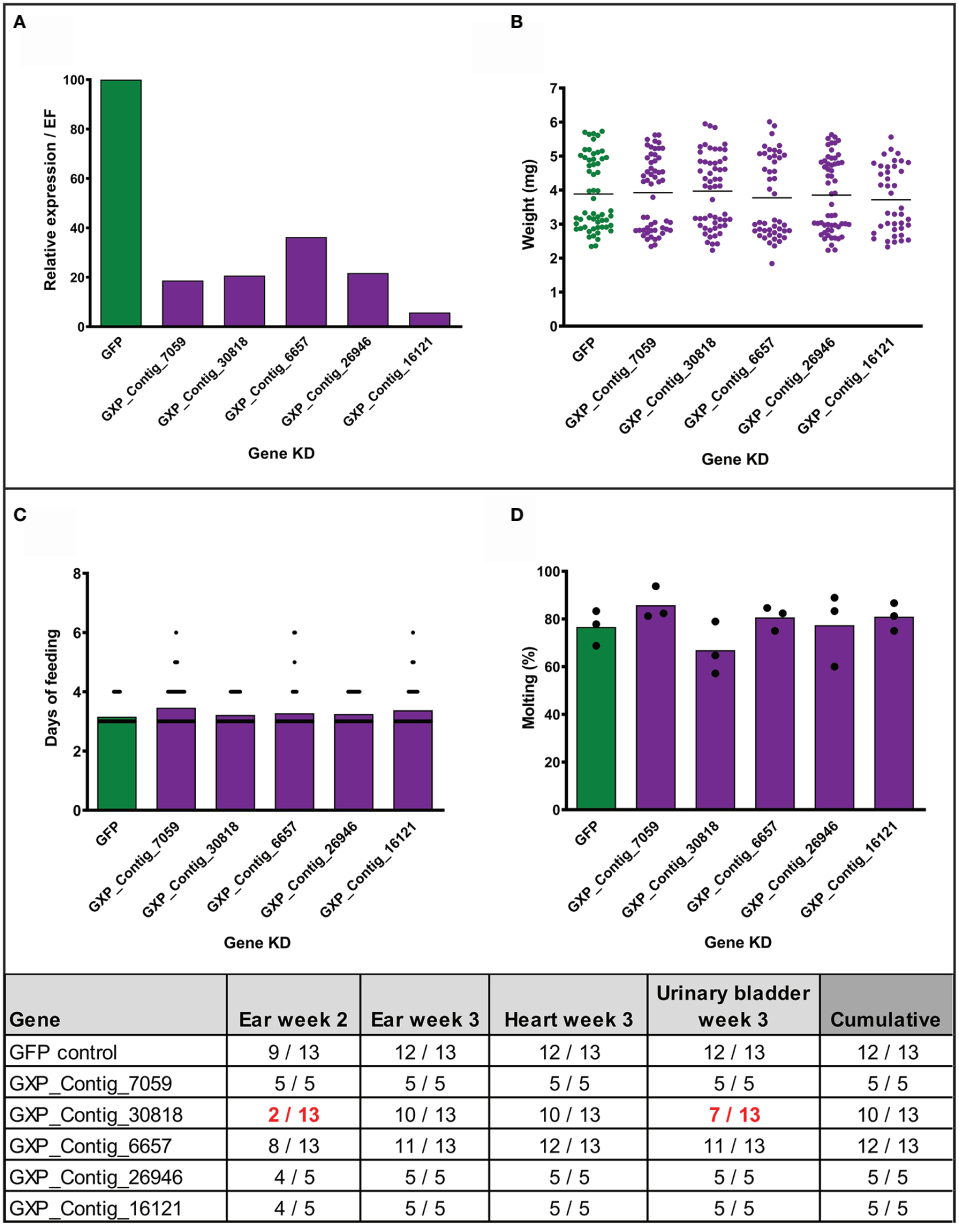
Effect of gene silencing by RNA interference on nymph feeding and *Borrelia afzelii* transmission. (Upper) Silencing of five tick genes in uninfected nymphs. **(A)** Evaluation of the silencing level by qRT-PCR (each group represents a mix of five fully fed nymphs). **(B)** Weights of individual fully fed nymphs. Each dot represents a single tick. **(C)** Duration of nymph feeding. **(D)** Molting success of fully fed nymphs into adults (percentage of molted nymphs fed on each mouse; biological triplicates). (Lower) Summary table of two transmission experiments with the gene-silenced *B. afzelii*-infected nymphs. Numbers indicate total qRT-PCR positive/total mouse tissues during the infection (ear week 2) and after mice scarification (week 3). dsGFP was used as a negative control. A decrease of positivity by >25% is highlighted in red.

To assess the role of the stimulated genes in transmission of *Borrelia*, we employed the method of RNA interference and injected nymphal ticks individually with five different gene-specific dsRNAs designed against the previously biologically validated transcripts. Before the transmission experiments with infected nymphs, we tested the effect of silencing in uninfected nymphs. The genes were successfully silenced in the fully fed nymphs to expression levels ranging from 6 to 36% comparing to the dsGFP control (**Figure 4A**). We did not observe any significant impact on feeding success, duration of feeding, tick weight after feeding, or molting of nymphs to adults (**Figures 4B, D**). We then performed the silencing in infected nymphs. Initially, we tested transmission with five mice per group. Silenced genes associated with the blocking of transmission of *B. afzelii* in at least one mouse, were further tested with an additional eight mice per group. Similarly, as observed with the uninfected nymphs, gene silencing did not affect tick feeding (**Supplemental Figures 3A, B**). The transmission of *B. afzelii* from the tick to the mouse was not noticeably blocked after the silencing of GXP_Contigs_7059, _6657, _26946, and _16121 (**Figure 4**). The number of spirochetes in deeper mouse tissues, as measured by qRT-PCR, was also not significantly altered (**Supplemental Figures 3C–F**). Interestingly, in the group with silenced uncharacterized protein (GXP_Contig_30818), the progress of infection in mice was delayed (only 15% of ears were *Borrelia*-positive by the second week compared to 70% in control), which was then reflected in a reduction of *Borrelia* prevalence in the ear (3^rd^ week), heart, and urinary bladder by 23, 23, and 46%, respectively (**Figure 4**).

## Discussion

The hypothesis that parasites actively modify the physiology and behavior of their hosts to enhance transmission is an intriguing and well-documented phenomenon in many species of living organisms (35). However, evidence of manipulation of ticks by *Borrelia* spirochetes is still mostly unknown. In this study, we have revealed differential gene expression in the midgut of *I. ricinus* nymphs infected with the Lyme borreliosis spirochete *B. afzelii* before, during, and after blood-feeding. This study represents the first transcriptome produced from ticks focusing on midgut genes stimulated by *Borrelia*. Previous transcriptomic studies described differential gene expression in salivary glands (33, 36), or used alternative approaches for such gene identifications (19, 20, 37–42).

The motile *Borrelia* enters the tick when larvae or nymph feed on an infected reservoir host. The spirochetes are attracted to the feeding site by the tick proteins secreted into the saliva (43). During the acquisition phase, the ingested spirochetes change their gene expression and multiply in the tick midgut contents (12, 44) to successfully infect the vector. After tick molting, the midgut appears empty. The midgut walls are localized close to each other, and the peritrophic matrix, a layer consisting of glycoproteins bound to the chitin network, is absent. In these harsh conditions of limited nutrients, which can last for months or years, the spirochetes switch into their “sleeping mode” and can be found attached to the midgut cell wall. OspA, a membrane lipoprotein produced by the *Borrelia*, was shown to bind the tick TROSPA protein present on the surface of *I. scapularis* midgut cells (19). *Trospa* was the first tick gene recognized as upregulated by the presence of *Borrelia* in the unfed nymph. Surprisingly, we were not able to identify *trospa* in our RefSeq database nor the recent TSA databases of *I. ricinus* available at NCBI. However, this gene has previously been sequenced from *I. ricinus* (NCBI: EU034646) (45), indicating that in *I. ricinus*, *trospa* was probably expressed to a limited level.

It is unknown how *Borrelia* spirochetes change expression of the tick midgut genes and how these modifications help *Borrelia* multiply and persist in the gut lumen. Using the MACE method on unfed midguts we have identified 210 downregulated and 165 upregulated tick genes as a result of infection (p-value ≤ 0.05 and log2 fold change ≤ −2.3 or ≥ 2.3). We found that *mitochondrial carboxypeptidase* (V5HK70) and *cytochrome C oxidase subunit VIa* (a component of the respiratory complex IV, XM_002435666) were downregulated > 8 fold in expression, indicating possible suppression of energy metabolism in unfed infected ticks. Among the highly upregulated genes, we identified several peritrophins and chitinases, constituents of the peritrophic matrix. However, the peritrophic matrix is formed in *I. ricinus* > 18h after the beginning of feeding (46), meaning that mRNAs of these genes could be pre-synthesized to accelerate the formation of the peritrophic matrix after the initiation of feeding. Alternatively, these proteins could be involved in establishing and maintaining other chitin structures such as tracheae, which supplement the midgut tissue with oxygen.

Ticks do not receive any nutrients from the environment, and the blood-feeding represents a significant milestone in their life cycles. The *Borrelia* spirochetes residing in the tick midgut become activated by a mechanism that is not completely clear [probably by nutrients in the blood, temperature, pH (47), osmolarity (48)] and thereby accelerate the expression of genes necessary for their transmission and survival in the vertebrate host. In the case of *B. burgdorferi* sensu stricto, the number of spirochetes multiplies from several hundred in an unfed nymph to a hundred thousand in a fully fed nymph (49). Next, *B. burgdorferi* migrate to the basolateral surface of the midgut epithelium, cross the basal membrane, and enter the hemocoel and salivary glands to infect the host through the secretion of saliva (14). However, *B. afzelii* appears to behave differently. These spirochetes do not multiply during feeding, but their numbers reduce continuously, possibly by direct traversal of the spirochetes from the midgut into the host (12). Importantly, spirochetes of *B. afzelii* have not been found to infect the salivary glands. In addition, and in contrast to *B. burgdorferi*, the number of *B. afzelii* spirochetes dramatically decreases over the next few months after molting (50). Bontemps-Gallo et al. previously showed that physicochemical parameters such as the level of oxygen, osmolality, and oxidative stress, affect growth and motility differently in these two genetically distinct bacterial species (51). Consistent with this, from 42 previously identified tick *Borrelia*-responsive genes (19, 20, 37–42) (including *tre31*, *isdlp*, *pixr*, *stat*, etc.), in our databases we found only *duox* (52) and *alcohol dehydrogenase* (42) being upregulated more than two-fold, further supporting the behavioral differences between *B. burgdorferi* and *B. afzelii*.

The primary purpose of this study was to identify tick proteins suitable for developing new anti-tick therapies. Ideally, such candidates should be abundantly expressed during feeding and targeted to the tick midgut wall or secreted from the cells into the midgut content in order to be accessible to antibodies or drugs present in the host blood. It was demonstrated that *B. afzelii* enters the host skin within 24h of attachment, but this population of spirochetes is not infectious. This means that the *Borrelia* need >24h for activation in the tick midgut to become infectious. We identified several tick genes altered in expression at the 24h time point. We do not know if this response was evoked explicitly by the *Borrelia* to gain an advantage during transmission, reflecting ongoing host modifications, or was induced by the tick as a reaction (immune) against the spirochetes.

We observed that at the fully fed time point, the number of upregulated genes were almost doubled when compared to the downregulated genes. We hypothesized that this overexpression was evoked by *Borrelia* during feeding to alter the tick physiology in order to transmit the spirochetes from the tick midgut into the host. To test the necessity of this upregulation, we silenced five previously biologically validated tick genes by RNA interference and tested the ability of nymphs to transmit *Borrelia*. All these candidates were predicted to be intracellular proteins, and many of them were transcription factors, so we tested whether silencing of these genes could block the expression of their downstream-regulated genes. We observed that the silencing of GXP_Contig_30818 caused the absence of *Borrelia* infection in the ear the second week after tick detachment (the beginning of infection) in 85% of mice (11 of 13). This delay in onset of disease probably triggered a further decrease in *Borrelia* prevalence in the ear (week 3) and destination tissues, heart, and urinary bladder. This transcript encodes yet uncharacterized protein with predicted nuclear localization. The expression of genes possibly regulated by this protein deserves further attention. The silencing of other genes did not affect *Borrelia* transmission. Therefore, we propose that upregulation of these genes is necessary for processes other than transmission, possibly for the acquisition and persistence of *Borrelia*. Additionally, in the transcripts upregulated during feeding, and similar to the unfed stage, we more often identified genes connected with synthesis and reconstruction of the peritrophic matrix (e.g., peritrophins and chitinases), whose expression has been previously shown to influence spirochete colonization of ticks (53).

We believe that this work will enable further identification and characterization of the tick midgut proteins necessary for acquisition, persistence, and transmission of *B. afzelii* from *I. ricinus*. In our MACE transcriptomic database, we found, in total, 55 *Borrelia*-stimulated, well expressed, and secreted or cell membrane-associated midgut proteins. We assume that some of these candidate proteins are necessary for *Borrelia* activation and transmission and that blocking of these proteins by a specific vaccine or a drug will contribute to the development of novel therapies against Lyme borreliosis.

## Data Availability Statement

The dataset presented in this study is deposited as **Supplementary Table 1** in the article/**Supplementary Material**.

## Ethics Statement

The animal study was reviewed and approved by the animal protection law of the Czech Republic (§17, Act No. 246/1992 Sb) with the approval of CAS (approval no. 79/2013).

## Author Contributions

RS, JT, PK, JH, and OH designed the experiments. SM, RS, VU, and OH prepared the infected nymphs, performed the dissections, isolated RNA, and accomplished the feeding and transmission experiments. NK and PW did the MACE analysis and annotations. SM analyzed the transcriptomic data and performed the technical and biological validations. SM and OH wrote the manuscript with an input from all co-authors. All authors contributed to the article and approved the submitted version.

## Funding

This work was supported by the Czech Science Foundation grant no. 20-05736S, the European Union FP7 project Antidote (grant agreement number 602272), and by the Centre for Research of Pathogenicity and Virulence of Parasites (no. CZ.02.1.01/0.0/0.0/16_019/0000759), funded by the European Regional Development Fund (ERDF) and Ministry of Education, Youth, and Sport, Czech Republic (MEYS).

## Conflict of Interest

NK and PW were employed by GenXPro GmbH.

The remaining authors declare that the research was conducted in the absence of any commercial or financial relationships that could be construed as a potential conflict of interest.

## Supplementary Material

Supplementary Figure 1 Scheme of sample preparation for biological validations. Ten populations of uninfected larvae, each originating from a single female fed on a guinea pig, were fed on *B. afzelii*-infected or uninfected mice. The nymphs then were fed on uninfected mice and dissected for midguts (10-50 nymphs for each group) at the three indicated time points.

Supplementary Figure 2
*Borrelia* infection does not affect tick feeding or final weights of the fully-fed nymphs. Each group of nymphs was comprised of females (higher weights) and males (lower weights). Each dot represents a single nymph. The data in each group contain a collection of 20 individual feedings (in total 360 infected and 339 uninfected nymphs). INF = infected nymphs, UNINF = uninfected nymphs. The horizontal bar indicates a mean. n.s. = not significant (Mann-Whitney test).

Supplementary Figure 3 Effect of gene silencing by RNA interference on nymph feeding and *B. afzelii* transmission.**(A)** Weights of individual fully-fed nymphs. Each dot represents a single tick. **(B)** Duration of nymph feeding. **(C–F)** The absolute number of *B. afzelii* in individual mouse tissues measured by qRTPCR. Two genes with no detectable *B. afzelii* in the heart tissue from the silencing Experiment 1 (left) were once more tested in the silencing experiment 2 (right). dsGFP was used as a negative control.

Supplementary Table 1 A list and expression of all Ixodes ricinus nymph midgut genes identified in individual MACE transcriptomes. UF = unfed, 24hrs = fed for 24 hours, FF = fully-fed, INF = B. afzelii-infected nymphs, UNINF = uninfected nymphs.

Supplementary Table 2 List of primers. Restriction sites for ApaI/XbaI are underlined.

Supplementary Table 3 Raw reads and mapped contigs obtained after the sequencing of MACE libraries. Raw reads mapped to our previously sequenced RefSeq library (Bioproject PRJNA657487) were labeled as GXP sequences. Sequences absent from the RefSeq library, but present in other Ixodes tick genomes and transcriptomes, were labeled as gi|. UF = unfed, 24hrs = fed for 24 hours, FF = fully-fed, INF = infected nymphs, UNINF = uninfected nymphs.

Supplementary Table 4 A list of Ixodes ricinus nymph midgut genes upregulated in the presence of Borrelia afzelii at three different timepoins of feeding. n.c. = not calculated.

Supplementary Table 5 A list of Ixodes ricinus nymph midgut genes co-upregulated by Borrelia afzelii at different timepoints. n.c. = not calculated.

Supplementary Table 6 A list of Ixodes ricinus nymph midgut genes downregulated in the presence of Borrelia afzelii at three different timepoins of feeding. n.c. = not calculated.

Supplementary Table 7 A list of Ixodes ricinus nymph midgut genes co-downregulated by Borrelia afzelii at different timepoints. n.c. = not calculated.
