# Supplementary figures and images for: Identification of Tick Ixodes ricinus Midgut Genes Differentially Expressed During the Transmission of Borrelia afzelii Spirochetes Using a Transcriptomic Approach
Source: Front Immunol. 2021 Feb 4;11:612412. doi: 10.3389/fimmu.2020.612412 (PMC7890033; doi:10.3389/fimmu.2020.612412)

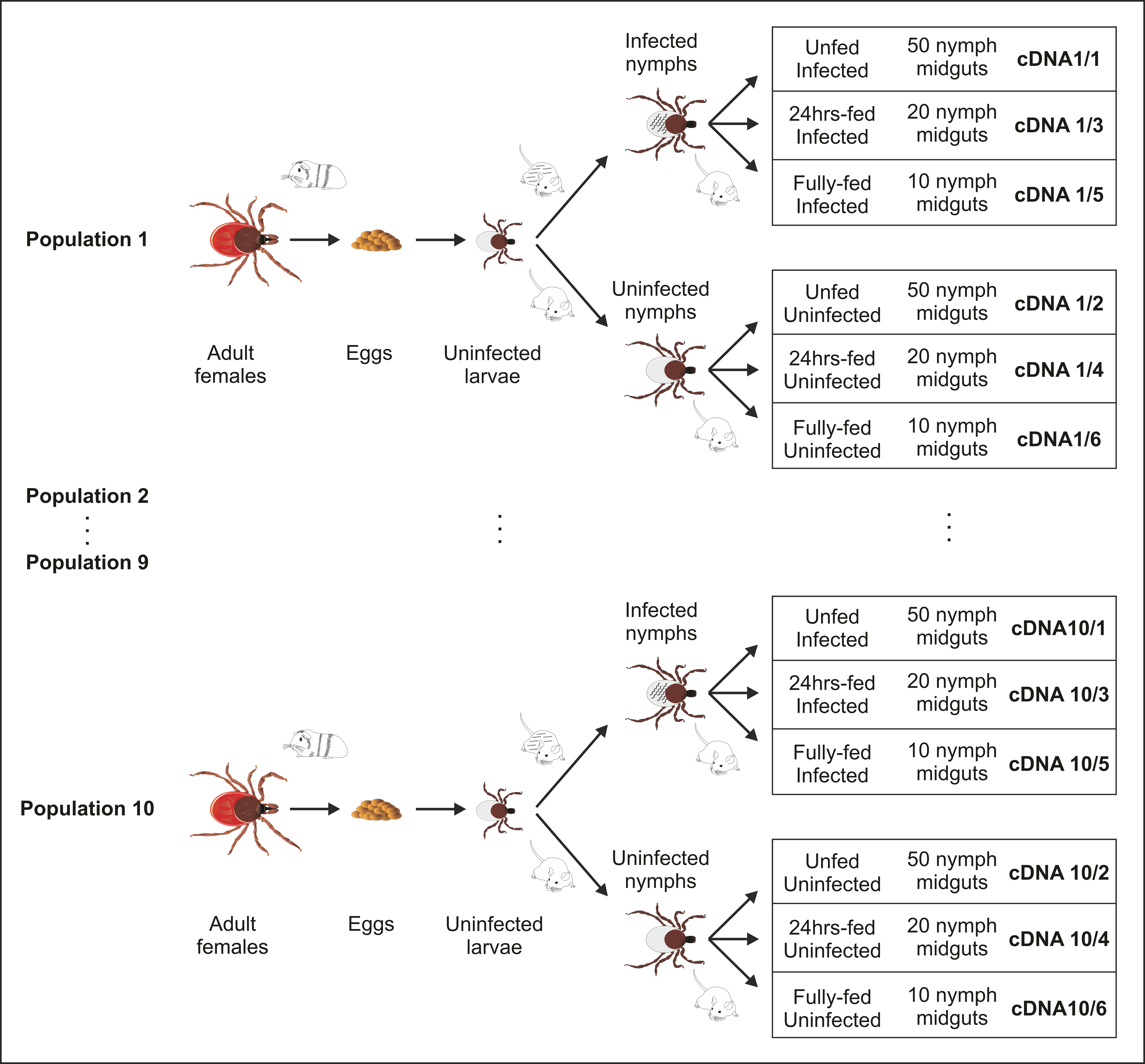

Supplement: Supplementary Figure 1 — Scheme of sample preparation for biological validations. Ten populations of uninfected larvae, each originating from a single female fed on a guinea pig, were fed on B. afzelii-infected or uninfected mice. The nymphs then were fed on uninfected mice and dissected for midguts (10-50 nymphs for each group) at the three indicated time points. [file Image_1.tif]

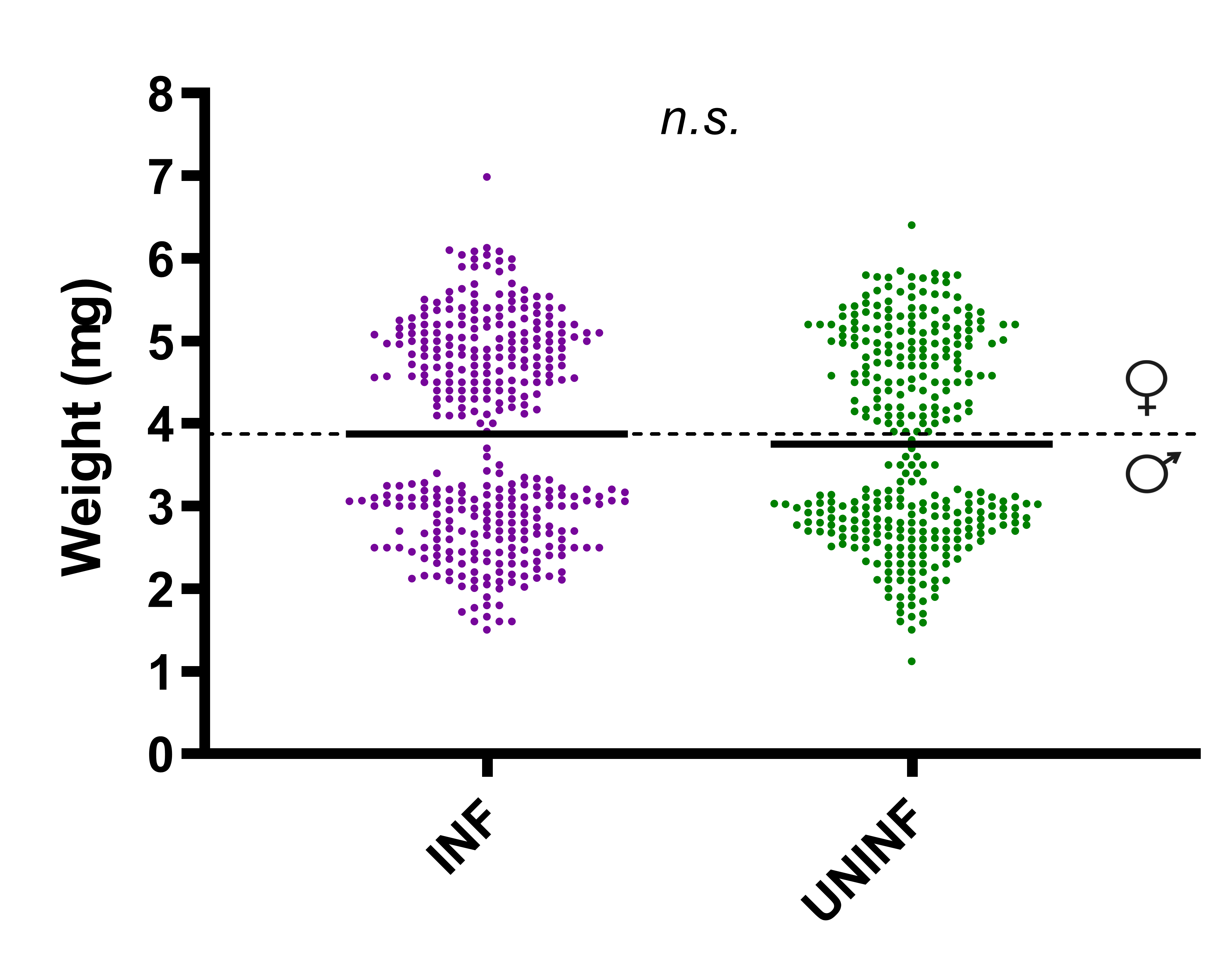

Supplement: Supplementary Figure 2 — Borrelia infection does not affect tick feeding or final weights of the fully-fed nymphs. Each group of nymphs was comprised of females (higher weights) and males (lower weights). Each dot represents a single nymph. The data in each group contain a collection of 20 individual feedings (in total 360 infected and 339 uninfected nymphs). INF = infected nymphs, UNINF = uninfected nymphs. The horizontal bar indicates a mean. n.s. = not significant (Mann-Whitney test). [file Image_2.tif]

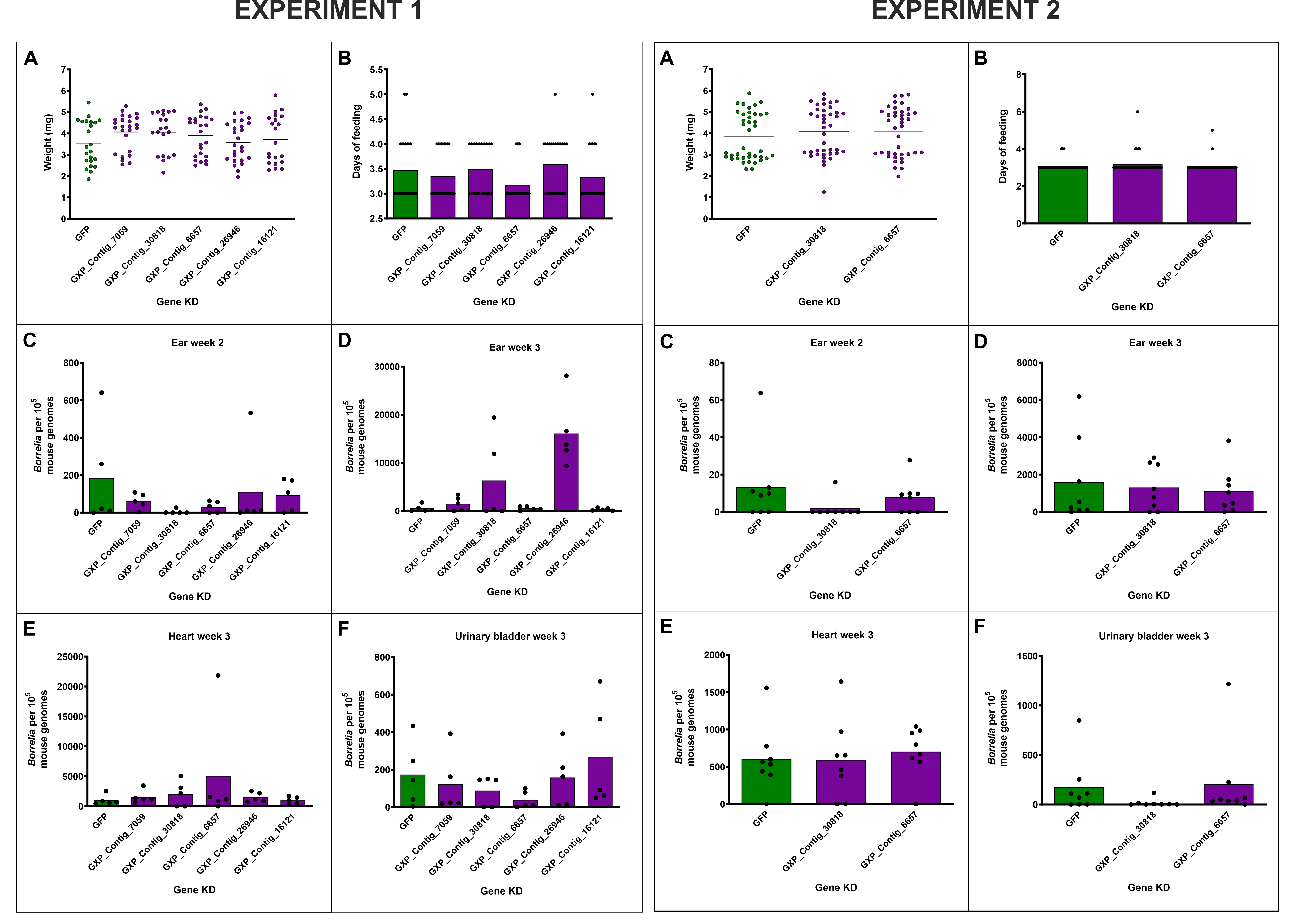

Supplement: Supplementary Figure 3 — Effect of gene silencing by RNA interference on nymph feeding and B. afzelii transmission.(A) Weights of individual fully-fed nymphs. Each dot represents a single tick. (B) Duration of nymph feeding. (C–F) The absolute number of B. afzelii in individual mouse tissues measured by qRTPCR. Two genes with no detectable B. afzelii in the heart tissue from the silencing Experiment 1 (left) were once more tested in the silencing experiment 2 (right). dsGFP was used as a negative control. [file Image_3.tif]
