# Supplementary material for: Identification of Tick Ixodes ricinus Midgut Genes Differentially Expressed During the Transmission of Borrelia afzelii Spirochetes Using a Transcriptomic Approach
Source: Front Immunol. 2021 Feb 4;11:612412. doi: 10.3389/fimmu.2020.612412 (PMC7890033; doi:10.3389/fimmu.2020.612412)
Supplement: Supplementary Table 2 — List of primers. Restriction sites for ApaI/XbaI are underlined. [file Table_2.docx]

# Supplementary Table 2 | List of primers. Restriction sites for ApaI/XbaI are underlined.

| **Method** | **Target** | **Name** | **Sequence (5´-->3´)** |
| --- | --- | --- | --- |
| RNAi | GXP_Contig_7059 | IR998 | ATGGGCCCTGGCGTCCTGAGCACTTTCG |
|  |  | IR999 | ATTCTAGAATGCACGAAAGAGACGATGG |
|  | GXP_Contig_30818 | IR996 | ATGGGCCCGAAGGGCGGGGAAAATGTGC |
|  |  | IR997 | ATTCTAGAAACAATTGAAATGCAGCCCG |
|  | GXP_Contig_6657 | IR988 | ATGGGCCCCGGTCAGTAGCTTGACCTCC |
|  |  | IR989 | ATTCTAGAAGGTCTCTTCGCTCCCATCG |
|  | GXP_Contig_26946 | IR1168 | ATGGGCCCCCTGCTCTCCTATCTCGTGG |
|  |  | IR1119 | ATTCTAGACTCGAATCCACTATATTGGG |
|  | GXP_Contig_16121 | IR990 | ATGGGCCCTGCAGTCGATCAGCAGCACC |
|  |  | IR991 | ATTCTAGATTTCCACACAGTTTTCATCC |
| qRT-PCR | GXP_Contig_7059 | IR788 | TAACTGCCGGGAGAGATAGC |
|  |  | IR789 | CTTTATTCGAGTCGCCCTTG |
|  | GXP_Contig_31514 | IR752 | GTAATCCGGCCAAATGACAC |
|  |  | IR753 | TTGCCCTAATACATGCACCA |
|  | GXP_Contig_30818 | IR756 | CTTGAGAATGTGGCACAACG |
|  |  | IR757 | ATCCACGCAAGTTGACACAA |
|  | GXP_Contig_5839 | IR754 | ACCTACGGCGACTACGCTAC |
|  |  | IR755 | TGAGGAAGTTGTTGGATGTGA |
|  | gi\|241123951\|ref\|XM_002403994.1\| | IR810 | AGCTACGGTGGACACTACGG |
|  |  | IR811 | ATCCTTCCGACAGCAGATCA |
|  | GXP_Contig_8911 | IR824 | CATGGTTTCGGAGGACACTT |
|  |  | IR825 | GCTTGTTCTTCCATGGTCGT |
|  | GXP_Contig_6131 | IR804 | GCCAAACGGTTTAACGACAT |
|  |  | IR805 | TATCGTTGGTGGTGTCCAGA |
|  | GXP_Contig_2889 | IR790 | ATATTGCAACACGACGCAAA |
|  |  | IR791 | TTATACTGCGGGGGTTTCAG |
|  | GXP_Contig_28121 | IR806 | AATATGCACAGGCTGCACAC |
|  |  | IR807 | TGCTACCAGAGGCCGAAATA |
|  | GXP_Contig_7109 | IR820 | CGCAGAAGAAGATCCAGGAG |
|  |  | IR821 | TCGATGAGTTGTTCGCACTC |
|  | GXP_Contig_30557 | IR822 | GTGCCCACAGTCTGATGAAG |
|  |  | IR823 | CCAGCTCACTTGCACCTTTA |
|  | GXP_Contig_16607 | IR890 | GCTGGTACAGAAAGGCTTCG |
|  |  | IR891 | ACGTCCTTCATCCACTCGAC |
|  | GXP_Contig_6657 | IR896 | ATCTACTTCGGGCGTGTTTG |
|  |  | IR897 | ACCTCATCCGTTTTCATTGC |
|  | GXP_Contig_25185 | IR886 | GCATCGGTTAAAATCGGAAA |
|  |  | IR887 | GCAAGCGGAATCTTCATTGT |
|  | GXP_Contig_26946 | IR892 | ACAACATAATCGGCCTGTCC |
|  |  | IR893 | ACCGTTGGGTGAAAACAAAA |
|  | GXP_Contig_476 | IR900 | GCATCACCCCACTCACTCTT |
|  |  | IR901 | GCTGATCCTTGTGGAGCTG |
|  | GXP_Contig_29696 | IR888 | CGAATTGTACAAGCGTCAGC |
|  |  | IR889 | CACTTTCACCTGGGCTGTTT |
|  | GXP_Contig_29976 | IR946 | GCTCATGTAGGGGAGCCATA |
|  |  | IR947 | CGCGGTTTTACAGTAGCACA |
|  | GXP_Contig_16121 | IR910 | TGCAGGAAATTCATGACACC |
|  |  | IR911 | CGCAAGCATAGGATCAGACA |
|  | GXP_Contig_21561 | IR748 | ACTTGGATGGAACGCTCAAG |
|  |  | IR749 | GCCGATGACCAAGGAGTTTA |
|  | GXP_Contig_1931 | IR758 | TGCCAAAAGACTCCGAACTT |
|  |  | IR759 | GCTAGGATCCTGCGTTCTTG |
|  | GXP_Contig_1305 | IR914 | TCCAGTGGAAGTTGAAGGAAA |
|  |  | IR915 | TGCCTTCTTAAGACGCGAAT |
|  | Tick *actin* | IR526 | cgacatcaaggagaagctctg |
|  |  | IR527 | gtcgggaagctcgtaggac |
|  | Tick *elongation factor* | IR524 | acgaggctctgacggaag |
|  |  | IR525 | cacgacgcaactccttcac |
|  | Tick *ferritin 1* | IR522 | gacttcctggagggcaacta |
|  |  | IR523 | attcggacagctccttgatg |
|  | Mouse *actin* (1) | MM-ACT-F | AGAGGGAAATCGTGCGTGAC |
|  |  | MM-ACT-R | CAATAGTGATGACCTGGCCGT |
|  |  | MM-ACT-PROBE | CACTGCCGCATCCTCTTCCTCCC |
|  | *Borrelia* spp. *flagellin* (2) | IR1345 | AGCAAATTTAGGTGCTTTCCAA |
|  |  | IR1346 | GCAATCATTGCCATTGCAGA |
|  |  | Fla Probe1 | TGCTACAACCTCATCTGTCATTGTAGCATCTTTTATTTG |
| PCR | *Borrelia* spp. *flagellin* (2) | IR1345 | AGCAAATTTAGGTGCTTTCCAA |
|  |  | IR1346 | GCAATCATTGCCATTGCAGA |

# REFERENCES

1. Dai J, Wang P, Adusumilli S, Booth CJ, Narasimhan S, Anguita J, Fikrig E. Antibodies against a Tick Protein, Salp15, Protect Mice from the Lyme Disease Agent. *Cell Host Microbe* (2009) **6**:482–492. doi:10.1016/j.chom.2009.10.006

2. Schwaiger M, Peter O, Cassinotti P. Routine diagnosis of Borrelia burgdorferi (sensu lato) infections using a real-time PCR assay. *Clin Microbiol Infect* (2001) **7**:461–469. doi:10.1046/j.1198-743x.2001.00282.x
