# Supplementary material for: Identification of Tick Ixodes ricinus Midgut Genes Differentially Expressed During the Transmission of Borrelia afzelii Spirochetes Using a Transcriptomic Approach
Source: Front Immunol. 2021 Feb 4;11:612412. doi: 10.3389/fimmu.2020.612412 (PMC7890033; doi:10.3389/fimmu.2020.612412)
Supplement: Supplementary Table 3 — Raw reads and mapped contigs obtained after the sequencing of MACE libraries. Raw reads mapped to our previously sequenced RefSeq library (Bioproject PRJNA657487) were labeled as GXP sequences. Sequences absent from the RefSeq library, but present in other Ixodes tick genomes and transcriptomes, were labeled as gi|. UF = unfed, 24hrs = fed for 24 hours, FF = fully-fed, INF = infected nymphs, UNINF = uninfected nymphs. [file Table_3.docx]

**SUPPLEMENTAL TABLE 3 | Raw reads and mapped contigs obtained after the sequencing of MACE libraries.** Raw reads mapped to our previously sequenced RefSeq library (Bioproject PRJNA657487) were labeled as GXP sequences. Sequences absent from the RefSeq library, but present in other *Ixodes* tick genomes and transcriptomes, were labeled as gi|. UF = unfed, 24hrs = fed for 24 hours, FF = fully-fed, INF = infected nymphs, UNINF = uninfected nymphs.

|  |  |  |  |
| --- | --- | --- | --- |
| **Library** | **Number of raw reads** | **GXP** | **gi\| (other *Ixodes* genomes and transcriptomes)** |
| MACE 1 (UF INF) | 4,765,625 | 17,261 | 1,255 |
| MACE 2 (UF UNINF) | 8,905,738 | 17,800 | 1,278 |
| MACE 3 (24hrs INF) | 9,248,649 | 18,179 | 1,354 |
| MACE 4 (24hrs UNINF) | 4,836,146 | 17,068 | 1,293 |
| MACE 5 (FF INF) | 5,826,650 | 17,757 | 1,363 |
| MACE 6 (FF UNINF) | 4,616,833 | 15,478 | 1,266 |
| **Total** | **38,199,641** |  |  |
|  |  |  |  |
|  |  |  |  |
